# The oral bioavailability of soil-borne risk elements for small terrestrial mammals: *Microtus arvalis* (Pallas, 1778) and *Apodemus sylvaticus* L. and its implication in environmental studies

**DOI:** 10.1007/s11356-023-26437-z

**Published:** 2023-03-21

**Authors:** Zuzana Čadková, Lenka Vořechovská, Denisa Javorská, Jiřina Száková, Pavel Tlustoš

**Affiliations:** 1 Department of Zoology and Fisheries, Faculty of Agrobiology, Food and Natural Resources, Czech University of Life Sciences, Kamýcká 129, CZ-165 21 Prague 6, Czech Republic; 2 Department of Agroenvironmental Chemistry and Plant Nutrition, Czech University of Life Sciences, Kamýcká 129, CZ-165 21 Prague 6, Czech Republic

**Keywords:** Common vole, Wood mouse, Risk element, Dietary intake, Soil ingestion, Bioindicator

## Abstract

The aims of this study were (i) to specify real risk elemetnt (RE) uptake by wild terresrial mammals (*A. sylvaticus* and *M. arvalis*), (ii) to describe RE distribution in critical organs such as the liver and kidney, and (iii) to determine potencial differences in RE toxicokinetics with regards to individual species or sex. Three groups of experimental animals were fed diets amended with soil and green biomass of hyperaccumulator *Arabidopsis halleri* with different RE levels. The contents of As, Cd, and Pb in the liver and kidneys of the animals reflected the element contents in the diet. Higher Cd and Pb accumulation ability was observed in *A. sylvaticus* compared to *M. arvalis* tissues, and an opposite pattern was recorded for As. Zn contents in tissues of both species remained unchanged, and total contents of Zn in the exposed animals even tended to decrease compared to the controls. Results of this study indicate a generally similar response of both species to elevated RE contents in the diet, confirming these wild rodents as suitable models for RE biomonitoring in ecosystems. However, our data highlights some distinction in As toxicokinetics in wood mice compared to that of field voles and a significantly higher accumulation of Pb and Cd in females. Therefore, factors of species and sex should not be overlooked if relevant data are to be obtained in environmental studies conducted on small terrestrial rodents.

**Supplementary Information:**

The online version contains supplementary material available at 10.1007/s11356-023-26437-z.

## Introduction

Chemical analyses of the contents of various pollutants in soil, water, air, etc. can provide useful information concerning the concentrations of these substances in the environment. However, information concerning the potential risk and toxicity of these pollutants for biota is limited. The use of animal specimens provide an in vivo perspective of RE consequences on wildlife. Comprehensive monitoring of animals living in contaminated areas, that can accumulate the pollutants in their tissues, can provide better information concerning the spatial or temporal changes of the environmental pollution in the ecosystems. These organisms can be used as bioindicators of environmental pollution (Michailova et al. 2010; Gall et al. 2015). For an estimation of the RE accumulation in animal organisms, various species have already been tested, comprising i.a. mollusks and passerine birds (Funes et al. 2006; Berglund et al. 2007). Among them, small terrestrial mammals (STM) showed a high ability to accumulate REs in their tissues due to their fast metabolism (Gall et al. 2015; González et al. 2008; Sánchez-Chardi et al. 2007; Tovar-Sánchez et al. 2012; Martiniaková et al. 2011). Results by Zhang et al. (2021) indicated high accumulation of toxic As in STM in typical grassland ecosysems. In species at second trophic level, such as rodents, Zn and As biomagnification occurs, while another group of STM such as insectivores at third trophic level had the strongest accumulation ability for Pb and As. Turna Demir and Yavuz (2020) proved vole specimen to be a suitable biological tool to monitor the extent of environmental pollution in areas contaminated due to mining activities. Regarding Czech Republic, *Microtus arvalis* has already been selected as a good bioindicator of soil contamination with Cd and Pb a few decades ago (Skácel and Pekárek 1992). Moreover, STM (including *Apodemus sylvaticus* and *M. arvalis*) are considered as suitable bioindicators not only for REs but also for organic soil contaminants such as polychlorinated biphenyls, polybrominated diphenyl ethers, and/or polyaromatic hydrocarbons (Vávrová et al. 2003; Marcheselli et al. 2010; Rodriguez-Estival and Smits 2016; Voorspoels et al. 2007). However, the recent review by Jota Baptista et al. (2022) has pointed out that not all animal tissues and organs provide the same information or should be interpreted in the same way. While non-invasive samples are more accessible and less stressful, invasive samples provide more detailed information with clinical and ecological relevance. In this context, the liver and kidney (such as the main organs that mediate detoxification) use to be the most frequently analyzed tissues.

Drouhot et al. (2014) showed a higher variability of As contents in the organs of STM compared to soil, indicating that As uptake by these animals is affected by additional variables other than the soil element content, where temperature, photoperiodism, location, animal species, dietary preferences, age, etc. should be taken into account (Viegas-Crespo et al. 2003; Fritsch et al. 2010). For instance, the RE contents in the skeleton of *Apodemus agrarius* are strongly age-dependent (Blagojevic et al. 2012). Although the REs are more easily absorbed via inhalation, dietary uptake of these elements is the predominant source for animals (Rogival et al. 2007). Such uptake comprises not only the RE directly incorporated in the target feed, but it may also be done by accidental ingestion of soil particles on the surface of animal or plant material (Garten 1980, Sheppard 1998). van den Brink et al. (2011) observed a difference in the response of Cd uptake and accumulation by *A. sylvaticus* and *M. arvalis* as related to their dietary preferences. They showed *A. sylvaticus* as a mobile species with a wide range in diet composition, whereas *M. arvalis* belong to the less mobile species consuming a diet from local sources. Therefore, Cd contents in the organisms of *A. sylvaticus* are related to the Cd contents in the consumed plants, whereas Cd contents accumulated in *M. arvalis* organisms are more related to the Cd contents and bioaccessibility in the soil. On the contrary, Martiniaková et al. (2012) compared the Pb, Zn, and Cd contents in the bones (femora) of *A. sylvaticus* and *M. arvalis* originating from different polluted areas, where no significant differences were recorded between the two species. Moreover, Beernaert et al. (2008) showed that *A. sylvaticus* individuals could select a diet with lower contents of REs compared to a more contaminated one. Similarly, Ozaki et al. (2018) showed decreasing preferences of RE-accumulating plants in the diet of *A. sylvaticus* with increasing soil and plant accumulation levels.

Accidental ingestion of soil was reported for many animal species and also for human beings. In areas characterized by low contents of nutrients in the soil–plant systems, soil ingestion could be an important source of these nutrients for grazing animals (Abrahams 2012). On the contrary, in the case of soil contaminated by either REs or organic pollutants, the ingestion of this soil can result in an increased uptake and accumulation of these pollutants in animal tissues with possible adverse effects on animal physiological and biochemical parameters comprising i.a. an increase of protein carbonyl levels and relative kidney weight or decrease of metabolic parameters such as body weight gain, food intake, water consumption, and urine and feces production (da Silva Jr. et al. 2013). Grazing herbivores living in contaminated areas can be exposed to organic and inorganic contaminants via consumption of contaminated pasture and/or via ingestion of contaminated soil. These aspects are well described in the case of livestock (Abad-Valle et al. 2018; Jurjanz et al. 2017; Chatelet et al. 2015), but information concerning the effect of contaminated soil consumption on STM is limited, although these animals are in permanent contact with the soil throughout their lives.

Significant correlations between RE contents in soil and RE contents in STM have been observed in numerous investigations (Shahsavari et al. 2019; Tête et al. 2014, Camizuli et al. 2018). Significant positive correlation among As, Cd, and Pb in soil-diet-*A. sylvaticus* tissue system and soil contamination gradient was confirmed by Rogival et al. (2007). However, the RE uptake and especially the uptake of soil-borne elements cannot be unambiguously estimated from the results of the monitoring provided in the contaminated areas. Therefore, the effect of a diet amended with soil contaminated with As, Cd, and Pb on the element uptake and physiological and hematological parameters was previously investigated in laboratory conditions using a rat model (Mascolo et al. 2004; Vlčková et al. 2014; Száková et al. 2012). However, the physiological and metabolic differences between laboratory strains of rodents (*Rattus* and *Mus*) and common wild species such as *Microtus*, *Apodemus*, and *Myodes* pose the pitfall of this methodological approach. Thus, knowledge concerning the potential effect of contaminated soil on the element accumulation in wild STM is still hampered by a lack of information concerning the real uptake rate of soil-borne elements by these animals.

Based on the facts mentioned above, we hypothesize that (i) controlled feeding study enables to determine the real uptake rate of soil-borne REs in wild STMs (bank voles and wood mice); (ii) RE concentrations in soft tissues of *M. arvalis* and *A. sylvaticus* will relevantly reflect the level of these elements in diet amended by contaminated soil; and (iii) the toxicokinetics of particular REs differ between both species and sexes due to certain differences in the biology. Thus, the main objectives of the present study were (i) to specify real RE uptake by selected species of wild small terresrial mammals (*A. sylvaticus* and *M. arvalis*), (ii) to desciribe RE distribution in their critical organs such as the liver and kidney, and (iii) to determine potencial differences in RE toxikokinetics with regards to individual species or sex. In order to simulate the normal diet of wild rodents as accurately as possible, we avoided adding an artifitial mixture of the individual elements to the compound feed, as was done in previous studies. Instead, the experimental diet was enriched directly with plant biomass and soil from the contaminated area. The replacement of model laboratory animals (rats) with selected wild animal species allows prediction of the actual response of STM to the enhanced RE contamination level, for instance in industrial areas.

## Material and methods

### Animals and experimental design

A total of 60 adult specimens of STM (39 individuals of *M. arvalis* and 21 individuals of *A. sylvaticus*) were trapped in the vicinity of the Sokolov brown coal basin (Northwest Bohemia, Czech Republic), divided according to sex, housed in cages in a room with a controlled temperature (22 ± 2 °C) under natural light conditions, and acclimatized for 2 weeks. During this period, one of the *Microtus arvalis* females showed signs of pregnancy; therefore, she was excluded from the further course of the experiment. Contaminated soils were collected in the vicinity of Příbram city (Central Bohemia, Czech Republic), an area with a long history of mining and smelting of Pb and Ag. The emissions from primary and secondary lead smelters resulted in high concentrations of REs (especially Pb, Cd, Zn and, to a lesser extent, As) in soils. Two locations were chosen for sampling: (a) a site next to mine waste heaps (49.710 N; 13.987 E), with a high RE background—referred to as HE—and (b) a site situated ca. 10 km from Příbram city (49.785 N; 13.987 E), with a lower RE background—referred to as LE.

Subsequently, animals of each species were divided into three experimental groups as follows: (i) control group (C) fed a standard feeding mixture with the addition of uncontaminated clover-grass fodder; a complete feed mixture for laboratory rodents was purchased from a commercial supplier (Velaz s.r.o., Czech Republic) and used as a control diet. The nutritional values, as declared by the manufacturer, are as follows: moisture—12.5%; nitrogenous compounds—24%; fiber—4.4%; fat—3.4%; ash—6.8%; lysin—14 g; methionin—4.8 g; Ca—11 g; P—7.2 g; Na—1.8 g; Cu – 20 mg; and Se – 0.38 mg DW; an uncontaminated clover-grass fodders were cultivated from grass seed mixture (Kiepenkerl) in pots under greenhouse conditions in the experimental facility of the Department of Agroenvironmental Chemistry and Plant Nutrition, and harvested biomass was continuously fed to experimental animals; (ii) the group labeled HE fed a standard feeding mixture amended by soil (10% w/w) from the HE site with the addition of *Arabidopsis halleri* biomass, a plant characterized by the hyperaccumulation ability for Cd and Zn, and originating directly from the HE site; and (iii) the group labeled LE with a standard feeding mixture amended by soil (10% w/w) from the LE site with the addition of *A. halleri* biomass originating directly from the LE site. Both variants (LE and HE) of *Arabidopsis halleri* plants were collected on particular localities, where LE and HE soil samples originated. Prior to the feeding study, several plant specimens were transported to the experimental facility of the Department of Agroenvironmental Chemistry and Plant Nutrition where they were cultivated in pots with LE and HE soil substrate. Fresh plant biomass was harvested and continuously fed to experimental animals along with pellets. In total, 114 ± 24 g of fresh *Arabidopsis halleri* biomass was addedd to the animal feed in the LE and HE groups during the feeding study. Individals in the control group recieved 139 ± 26 g of fresh clover-grass biomass for the same period. The 10% w/w rate of soil was chosen according to our previous experiments with a laboratory rat model (Vlčková et al. 2014, Száková et al. 2012). The element contents in the individual components of the diets are summarized in Table 1.

**Table 1:** The total element contents in the experimental soils, forage, and feeding mixtures

	As	Cd	Pb	Zn
	mg/kg			
Control pellets	a	0.031 ± 0.001	a	94.9 ± 4.9
Soil LE	18.5 ± 9.5	1.61 ± 0.29	241 ± 46	274 ± 78
Soil HE	629 ± 92	43.9 ± 26.3	3898 ± 329	6310 ± 1725
Pellets+soil LE	10.8 ± 1.2	1.03 ± 0.20	86.3 ± 2.2	191 ± 2.5
Pellets+soil HE	61.4 ± 0.3	4.22 ± 0.02	446 ± 28	759 ± 66.2
Clover-grass forage	0.035 ± 0.003	0.009 ± 0.005	0.331 ± 0.179	39.8 ± 0.1
*A. halleri* LE	2.42 ± 0.05	41.1 ± 0.6	39.8 ± 0.4	6689 ± 132
*A. halleri* HE	17.3 ± 1.9	66.4 ± 6.6	117 ± 17	11170 ± 737
Preventive values for soil*	20	0.5	60	120
Indicative values for soil*	40	20	400	-
Maximum limit for feed mixture**	2	0.5	5	-
Maximum limit for raw forage**	2	1	30	-

Data are presented as average ± standard deviation

*a* data under detection limit

*According to Public notice No. 153/2016 for the protection of agricultural soil quality in the Czech Republic, **according to Directive No. 2002/32/ES of European Parliament and Council of Europe concerning xenobiotics in feedstuffs

For 7 weeks, the animals were fed ad libitum, and the feed consumption was regularly monitored. A composite sample of animal feces was collected weekly from each group. At the end of the experiment, the animals were euthanized by exsanguination after inhalation anesthesia. Liver and kidneys were sampled, properly washed in deionized water, stored at −80 °C, freeze-dried, and homogenized prior to subsequent chemical analyses.

### Analytical methods

The pseudo-total contents of elements in the soils were determined in the digests obtained as follows: aliquots (~0.5 g) of air-dried soil samples were decomposed in a digestion vessel with 10 mL of aqua regia (nitric and hydrochloric acid mixture in a ratio of 1:3). The mixture was heated in an ETHOS 1 (MLS GmbH, Germany) microwave-assisted wet digestion system for 33 min at 210 °C. An aliquot (~500 mg of dry matter) of the homogenized sample was weighed in a digestion vessel for determination of the elemental contents in the *A. halleri* plant biomass, clover-grass fodder, unamended feeding mixture, soil-amended pellets, and homogenized excrement samples. Concentrated nitric acid (8.0 mL) (Analytika Ltd., Czech Republic) and 30% H_2_O_2_ (2.0 mL) (Analytika Ltd., Czech Republic) were added. The mixture was heated in an ETHOS 1 (MLS GmbH, Germany) microwave-assisted wet digestion system for 30 min at 220 °C. After cooling, the digests were quantitatively transferred into a 25-mL glass tube, topped with deionized water, and kept at laboratory temperature until measurements were taken. Inductively coupled plasma-optical emission spectrometry (ICP-OES, Agilent 720, Agilent Technologies Inc., USA) was used for the determination of As, Cd, Pb, and Zn contents in the digests, where the following experimental conditions were used: power of 1.2 kW, plasma gas flow of 15.0 L/min, auxiliary flow of 0.75 L/min, and nebulizer flow of 0.9 L/min.

The freeze-dried and homogenized animal tissue samples were decomposed in a microwave-assisted wet digestion system with focused microwave heating (Discover SPD-Plus, CEM Inc., USA). An aliquot (~0.5 g of dry matter) of the tissue sample was weighed in a quartz-glass digestion vessel (35 mL volume). Then, 10 mL of concentrated ANALPURE® nitric acid (Analytika Ltd., Czech Republic) was added; the mixture was heated at a maximum power of 300 W, at 202 °C, and 21 bar for 8 min. After cooling, the solution was quantitatively transferred to plastic tubes, filled to 50 mL with deionized water, and kept at laboratory temperature until measurement. The As, Cd, Pb, and Zn contents of the digests were measured by inductively coupled plasma mass spectrometry (ICP-MS, Agilent ×7700, Agilent Technologies Inc., USA) using a collision cell to decrease potential spectral interferences. The operating conditions were as follows: RF power of 1550 W, sampling depth of 8 mm, plasma gas flow of 15.0 L/min, auxiliary flow of 0.9 L/min, and helium collision cell flow of 8 L/min. An ASX-500 autosampler, a three-channel peristaltic pump, and a MicroMist nebulizer were used for the ICP-MS measurement.

### Data processing

The obtained dataset was processed using Statistica 12 Cz software (StatSoft. Inc. software). Differences in tissue RE concentrations among the three experimental groups (C, LE, and HE) were tested by one-way ANOVA with post hoc comparisons using the Tukey HSD method. Subsequently, the differences in mean RE concentrations between male and female specimens were tested using two-sample *t*-tests within each experimental group. Differences in RE excretion dynamics were tested using a repeated measures ANOVA, where “week” was used as a nested factor for each experimental group. A criterion of *α* = 0.05 was used for significance in all applied tests. Subsequently, potential correlations among particular RE tissue concentrations in both rodent species were tested.

The exact portions (%) of the RE dose accumulated in the animal tissues or excreted from their bodies were determined based on the defined RE amount (mg) received by each experimental group, tissue RE concentrations (mg/kg), specific dry weights of liver and kidneys (kg) of each specimen, or total dry weight (kg) of the feces produced by each experimental group for the whole experiment. Additionally, the final element contents of both exposed groups (LE and HE) were standardized to those of the control group, which were considered as background levels.

## Results and discussion

### The RE contents in the feeding mixtures and soils

The public notice characterizing the conditions for the protection of agricultural soil quality in the Czech Republic (Public notice No. 153/2016) was used as a basis for assessment of the RE levels in the soils. The Cd, Pb, and Zn concentrations in the LE soil just exceeded the preventive values (0.5 mg/kg for Cd, 60 mg/kg for Pb, and 120 mg/kg for Zn), and As was very close to this limit—20 mg/kg for As; the RE contents in the HE soil pose not only a direct risk to crop contamination and soil biota but can directly threaten animal and human health (exceeding the indicative values of 40 mg/kg for As, 20 mg/kg for Cd, 400 mg/kg for Pb) presented in Table 1.

While the RE contents in the control feeding pellets were low, the element contents in both variants of the soil-amended diet exceeded the maximum permissible limits for complete feeding mixtures (Directive No. 2002/32/ES), representing 2 mg/kg for As, 0.5 mg/kg for Cd, and 5 mg/kg for Pb (Table 1). According to this directive, the maximum permissible limits for raw forage are 2 mg/kg for As, 1 mg/kg for Cd, and 30 mg/kg for Pb. Therefore, the control clover-grass forage met these requirements, whereas the element contents in the *A. halleri* biomass showed elevated contents of the elements, exceeding the limits, while the differences between the *A. halleri* biomass were related to the different element contents in the soils (Table 1). The results confirmed the Cd and Zn accumulation ability of *A. halleri* (Pollard et al. 2014), but the high contents of As and Pb in the soil also resulted in enhanced levels of these elements in plants. Although the As and Pb levels in *A. halleri* did not reach the criteria for element hyperaccumulation ability (1000 mg/kg, according to van der Ent et al. 2013), the levels of these elements in the plant biomass exceeded the maximum permissible limits for raw forage (see above).

### The RE contents in the animal tissues and feces

**Fig. 1:**
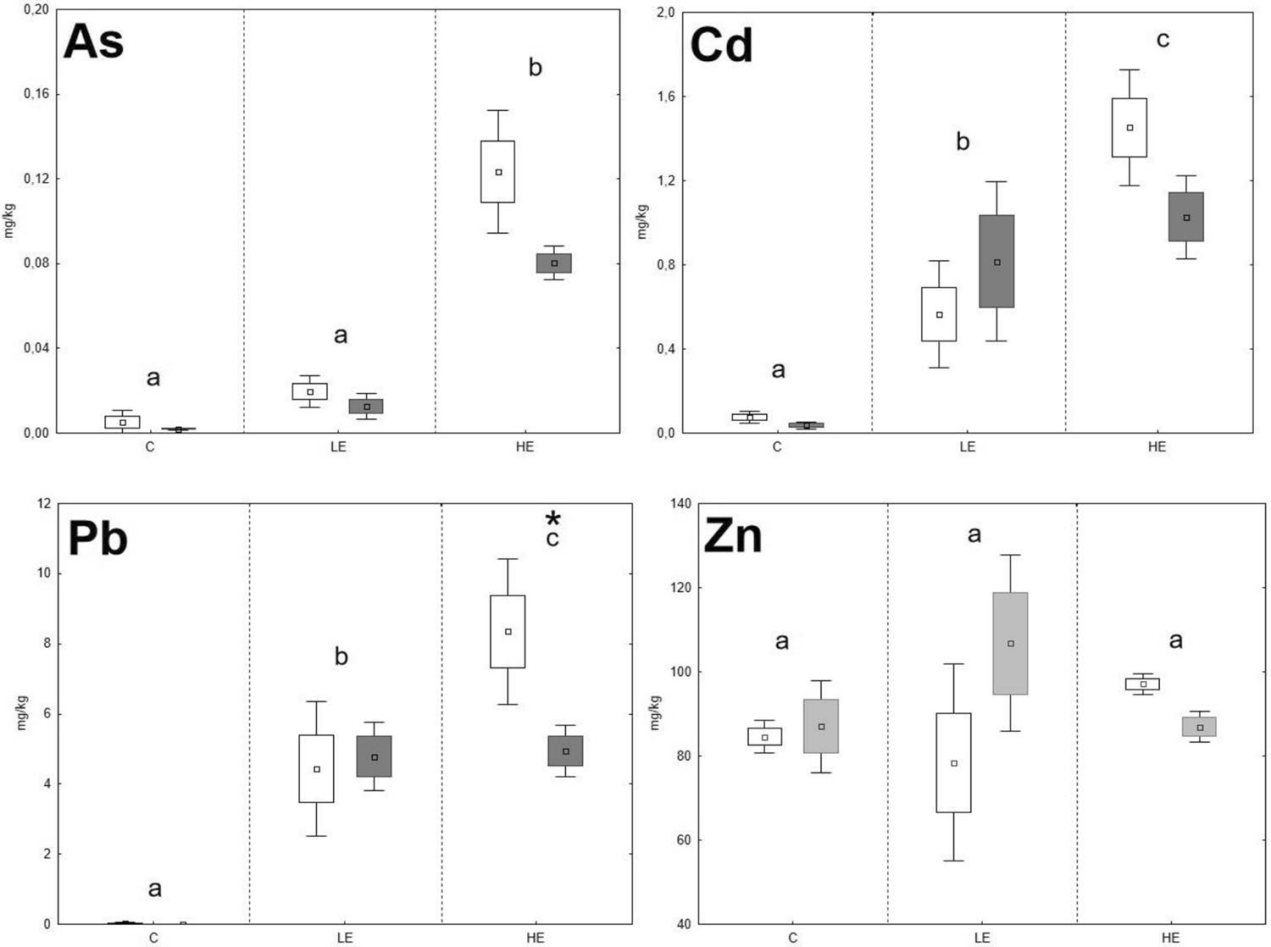
The element contents in the liver of *A. sylvaticus*; data expressed as mean (point), standard error (box), and 95% confidence interval (line segment); the boxes marked by the same letter did not significantly differ at *p* < 0.05 within individual groups regardless of sex; the boxes marked by * indicate significant difference between males (grey box) and females (white box) within one group

**Fig. 2:**
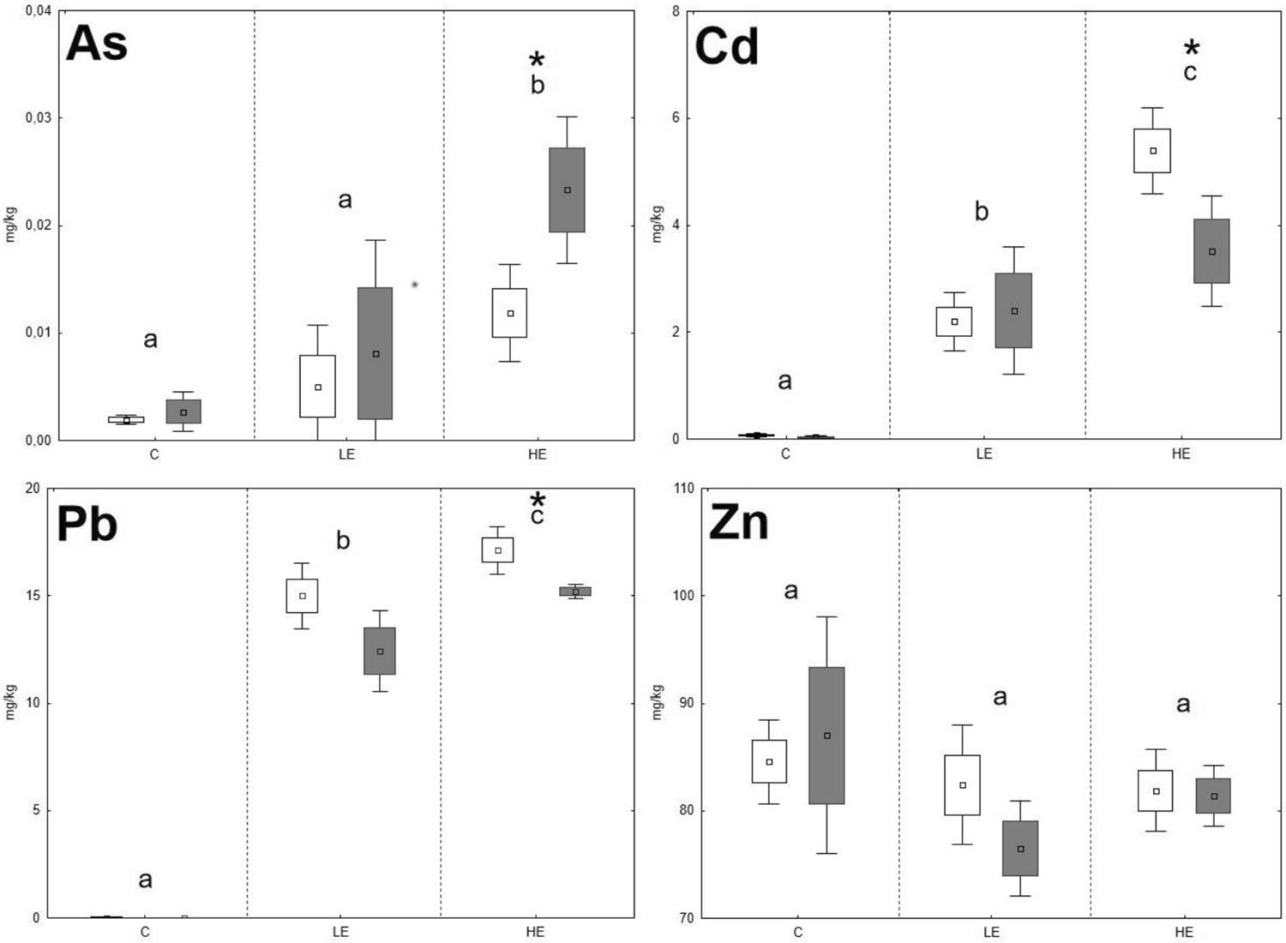
The element contents in the kidney of *A. sylvaticus*; data expressed as mean (point), standard error (box), and 95% confidence interval (line segment); the boxes marked by the same letter did not significantly differ at *p* < 0.05 within individual groups regardless of sex; the boxes marked by * indicate significant difference between males (grey box) and females (white box) within one group

**Fig. 3:**
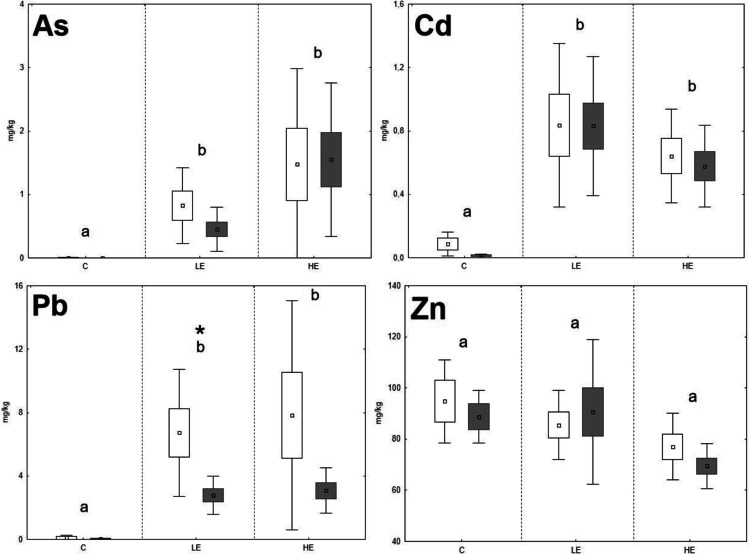
The element contents in the liver of *M. arvalis*; data expressed as mean (point), standard error (box), and 95% confidence interval (line segment); the boxes marked by the same letter did not significantly differ at *p* < 0.05 within individual groups regardless of sex; the boxes marked by * indicate significant difference between males (grey box) and females (white box) within one group

**Fig. 4:**
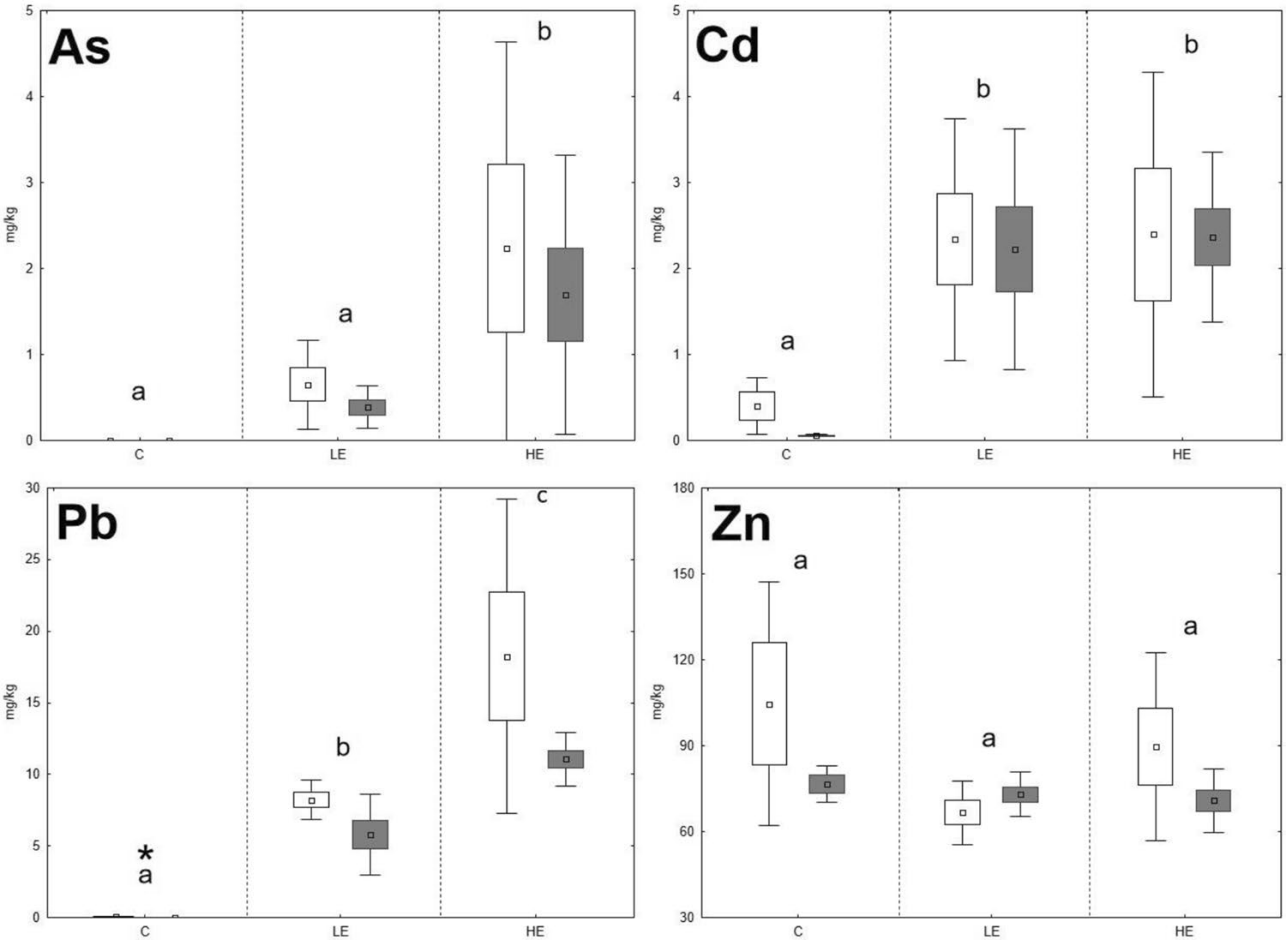
The element contents in the kidney of *M. arvalis*; data expressed as mean (point), standard error (box), and 95% confidence interval (line segment); the boxes marked by the same letter did not significantly differ at *p* < 0.05 within individual groups regardless of sex; the boxes marked by * indicate significant difference between males (grey box) and females (white box) within one group

The As and Pb contents in the liver and kidneys of both animal species increased significantly with the increasing content of these elements in the diet (Figs. 1, 2, 3, and 4). However, a higher accumulation ability of As was observed for *M. arvalis*, where the As contents in the liver and kidneys of the HE group reached up to 1.52 ± 0.23 mg/kg and 1.91 ± 0.32 mg/kg, respectively, whereas the As contents in the liver and kidneys of *A. sylvaticus* in the HE group were 0.11 ± 0.01mg/kg and 0.017 ± 0.002 mg/kg, respectively. In the case of Pb, the element contents in the liver and kidneys of both species were similar, where the Pb contents in the liver and kidneys of *M. arvalis* in the HE group were 5.30 ± 1.04 mg/kg and 13.9 ± 1.3 mg/kg, respectively, and the Pb content in the liver and kidneys of *A. sylvaticus* in the HE group was 6.89 ± 0.61 mg/kg and 16.3 ± 0.5 mg/kg, respectively. As expected, Pb concentrations measured in the livers and kidneys of all exposed groups in the present experiment significantly exceeded levels recorded by various authors in the soft tissues of free-living arvicolid and murid rodents (Ma, 1989, 1991; Petkovšek et al. 2014, 2015, 2017). Moreover, several soft tissue Pb concentrations in specimens fed high-contaminated diet exceeded the lowest effect concentration for the wild rodents as reported by Ma (1996). According to this author, the kidney and liver Pb levels higher than 15 and 5 mg/kg DW, respectively, may be associated with structural and functional kidney damage.

The significant stepwise increase of Cd in animal organs with increasing Cd content in the diet was proven only in the case of *A. sylvaticus*, where the Cd contents in the liver rose in the order of 0.06 mg/kg (C), 0.67 mg/kg (LE), and 1.27 mg/kg (HE), respectively; and a similar trend was observed in the case of the kidneys, where the Cd contents increased in the order of 0.06 mg/kg (C), 2.28 mg/kg (LE), and 4.59 mg/kg (HE), respectively. On the contrary, the Cd levels in the liver and kidneys of *M. arvalis* increased significantly (*p* < 0.05) in the LE group compared to the C group, but in the HE group, the Cd levels did not increase compared to the LE group and even tended to decrease in the liver. Fritsch et al. (2010) reported a high ability of metallothionein synthesis in the *A. sylvaticus* (murid rodent) organism compared to *Myodes glareolus* (arvicolid rodent). It could be speculated that higher Cd contents in the *A. sylvaticus* liver may be related to their immobilization in the metallothionein molecular structure during high exposure to this RE. For Zn, no significant changes of element contents related to the increasing Zn levels in the diet were observed. The Zn levels in the kidneys of both species even tended to decrease in the LE and HE groups compared to the C group. These results confirm the ability of animal organisms to achieve a homeostasis of Zn as an important essential element regardless of the high content of this element in the diet (Jančová et al. 2006; Prasad 2008). Additionally, competition with REs during uptake, absorption, and deposition may reduce the final concentrations of essential elements in animal tissues (Whittaker et al. 2011; Wang et al. 2009). For instance, decreasing Zn levels in the organism exposed to elevated rates of Cd was published by Matovic et al. (2011), and similar pattern was observed in our previous experiments with rat model (Vlčková et al. 2014).

In order to detect potential interaction among administered REs, we tested the correlation among particular tissue concentrations in both rodent species. In animals fed control and low-exposure diet, the correlations among the RE concentrations showed just inconsistent pattern. However, with high RE intake, several correlations become more evident. Concerning high exposed *Apodemus sylvaticus*, concentrations of all measured REs increased, while As concentration in the kidney decreased (*r*^2^ up to −95%). On the contrary, strong positive correlation were found between liver Zn and As (*r*^2^ = 70%), liver Zn and Pb (*r*^2^ = 78%), liver and kidney Pb (r^2^ = 78%), liver and kidney Cd (*r*^2^ = 80%), and liver Cd and Pb in both soft tissues (*r*^2^ = 82 and 83%). Regarding *Microtus arvalis*, statistical analyses revealed negative correlation between concentration of arsenic and those of Zn, Cd, and Pb (*r*^2^ up to −64%). Positive correlation coefficients were demonstrated in this species among all other elements, the highest between Zn and Pb (*r*^2^ = 70–80%), Zn in the liver and kidney (*r*^2^ = 74%), and Pb in the liver and kidney (93%). Summary of particular relationships is shown in correlation plots (Suplementary figure S2).

Whereas the fate of individual REs in the animal organism is well documented, the findings in the case of RE interactions in the animal tissues are very limited and often contradictory. The different response of the animal organism on the co-exposure to the REs depends on (i) the chosen element mixture, (ii) the chemical form of the elements applied, (iii) pollution level, (iv) particular tissue exposed, and (v) duration of the experiment (Cobbina et al., 2015, Alonso et al. 2002). Among the possible inter-element interrelationships, the negative relations between Cd and As were most frequently investigated. In this context, Száková et al. (2009) found out that the the addition of As to the rat diet decreased the accumulation of Cd in the kidney and increased its accumulation in the testes, and on the contrary, the addition of Cd to the diet increased arsenic content in the liver and kidney and decreased its content in the testes. Similarly, Ollson et al. (2017) stated that Cd co-exposure with As decreased the bioavailability of As, and Cd co-exposure with Pb increased the accumulation of Pb in the liver. They also explained the As-Cd interrelationships by the Cd influence on the arsenate absorption as a consequence of the impairment of phosphate transporters.

The bioavailability of soil-borne elements depends on the characteristics of the soil (soil pH, element species in the soil, element binding on the soil particles, etc.), frequency of the animal contacts with the contaminated soil, and also on the animal characteristics such as sex, age, and body weight (Camizuli et al. 2018). Moreover, substantial changes in the mobile proportions of elements can occur in the digestion tract due to the reactions of the soil particles and other components of the diet with the gastric and intestinal fluids (Yang 2010). The response of the animal organisms on the soil-bearing elements as related to the physicochemical parameters of the soils presented Vlčková et al. (2014) for the rat model. In their experiment, the bioavailability of the elements decreased in order Cd > As > Pb. Similarly, Mascolo et al. (2004) investigated the bioavailability of REs (including As, Cd, and Pb) contained in various clay materials by using a rat model, where element contents in the liver and kidneys of rats increased, and the order of element bioavailability was also Cd > As > Pb. Lower bioavailability of Pb compared to As was also noted by Ellickson et al. (2001).

Although a different method was used for RE bioavailability evaluation (based on total portions (%) of the RE dose accumulated in the tissues) in the present study, our findings partially comply with the abovementioned studies. We found a five-fold higher accumulation ability for Cd (0.3‰) compared to that of Pb (0.06‰), and two-fold higher ratios of Pb when compared to As (0.03‰). Moreover, significant differences were observed between two experimental organisms. Whereas relatively balanced contents of monitored RE were detected in an arvicolid herbivore, *M. arvalis*, a murid omnivore, *A. sylvaticus*, showed a higher accumulation capacity for Cd and an extremely low As ratios. The overall RE ratios stored in rodent livers and kidneys were negligible. On the other hand, approx. 37%, 32%, 44%, and 27% of As, Cd, Pb, and Zn, respectively, were excreted from their body via feces (Table 2). For Zn, the results showed equal or even lower total amounts of Zn in both the liver and kidneys of the HE and LE groups compared to the control, indicating a lower proportion of the accumulated Zn in the HE and LE groups. In this case, the potential Cd–Zn competition could be speculated as one possible explanation. Generally, this method of bioavailability evaluation demonstrates that an extremely high RE intake leads to lower rates of accumulation and higher rates of element elimination, whereas a higher portion of the administered dose can be stored in the body, and RE excretion is not so intensive during lower RE exposure.

**Table 2:** Element balance in experimental animals

Species	Group	Total element intake (mg)				A portion of the element dose excreted via faces (%)*				A portion of the element dose stored in the tissue (%)*				
		As	Cd	Pb	Zn	As	Cd	Pb	Zn		As	Cd	Pb	Zn
*Apodemus sylvaticus*	LE	2.78	0.85	21.8	126	32	29	53	28	Liver	< 0.001	0.035	0.010	0.002
										Kidney	< 0.001	0.024	0.006	< 0.001
	HE	14.3	1.56	105	267	59	49	61	41	Liver	< 0.001	0.045	0.004	0.002
										Kidney	< 0.001	0.027	0.001	< 0.001
*Microtus arvalis*	LE	3.50	1.44	27.4	225	26	22	33	16	Liver	0.014	0.039	0.013	a
										Kidney	0.002	0.014	0.003	a
	HE	19.7	2.19	144	350	32	28	30	24	Liver	0.008	0.022	0.003	a
										Kidney	0.001	0.011	0.001	< 0.001

*a* lower compared to control group

*The final element contents were standardized to those of the control group, which were considered as background levels

The relationship of the RE contents in the animal tissues and the contents of these elements in soil was also identified by other authors. For instance, a series of studies by Al Sayegh Petkovsek et al. (2014, 2015 and 2017), that dealt with the correlation of REs between soil and animal tissue at locations with different sources of pollution (a former lead smelter, a busy motorway, and a thermal power plant), confirmed the use of small mammals (first of all *M. glareolus* and *A. flavicollis*) as suitable biomonitors of metal pollution. Results of present experiment indicate a higher availability of Pb compared to As, suggesting higher accessibility of Pb in an area affected by long-term emissions from a Pb smelter. Milton and Johnson (1999) determined the As contents in small terrestrial herbivores living in an area affected by former mining activities, where the As contents in the mine tailings reached up to 630 mg/kg, i.e., a similar value to the soil in the HE group. In line with our results, these authors found a low level of As in both plant biomass and animal tissues, confirming the low bioavailability of As in the soil–plant–animal food chain.

Age, sex, and nutritional status of animals can affect RE accumulation in individual organs (González et al. 2008). Previous studies confirmed changing RE accumulation ability as being related to the age and sex of *A. sylvaticus* (Zarrintab and Mirzaei 2017; Sánchez-Chardi et al. 2007), where these elements were more intensively accumulated in young animals, due to their higher nutrient requirements and therefore higher dietary intake. The age-dependent element uptake by animals could be speculated as being one of the possible reasons for the high variability of our results, as the animals caught in natural conditions were not exactly of the same age. On the contrary, the potential effect of sex was considered in this study, although no significant effect of sex on the Pb and Zn accumulation in the liver and kidneys of *A. sylvaticus* was previously published (Tifarouine et al. 2019). Concerning the present study, the element contents in the livers of males tended to lower values compared to females in most of cases, where a potential unambiguous effect was hampered by the high variability of the data (Figs. 1, 2, 3, and 4). A significant (*p* < 0.05) effect of sex was recorded for both liver and kidney Pb contents and kidney Cd contents of *A. sylvaticus*. An opposite pattern was observed in the case of As in *A. sylvaticus* kidneys (Fig. 2). Significant (*p* < 0.05) differences for As, Cd, and Pb were shown only in the case of the HE groups. Concerning *M. arvalis*, the higher RE concentrations were also generally detected in females. However, a significant difference was shown just for Pb in the LE group. Thus, the sex differences in RE uptake were more apparent in the case of *A. sylvaticus* compared to *M. arvalis* and occurred mainly during elevated RE exposure.

**Fig. 5:**
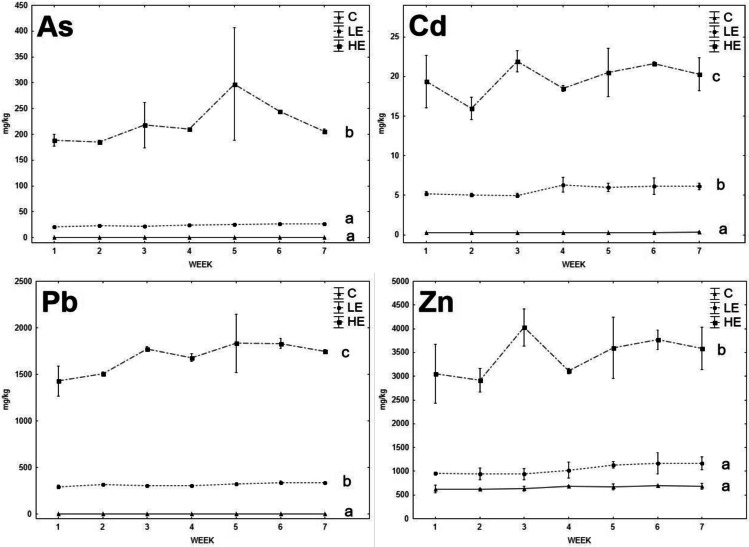
The element excretion dynamics via feces in *A. sylvaticus*; data expressed as mean (point) and standard error (line segment); the lines marked by the same letter did not significantly differ at *p* < 0.05 within individual groups regardless of sex

**Fig. 6:**
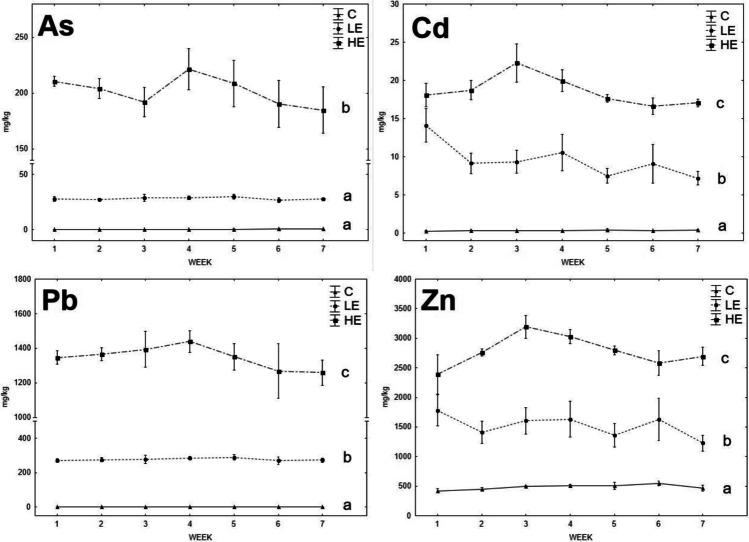
The element excretion dynamics via feces in *M. arvalis*; data expressed as mean (point) and standard error (line segment); the lines marked by the same letter did not significantly differ at *p* < 0.05 within individual groups regardless the sex

Figures 5 and 6 summarize the RE contents in the weekly sampled feces of the animals. The element contents in the feces reflected the contents of these elements in the diet including Zn, which was balanced in the liver and kidneys regardless of the Zn contents in the diet (Figs. 1, 2, 3, and 4). The RE contents in the feces of *A. sylvaticus* showed significantly (*p* < 0.05) increasing contents of Cd and Pb in the order of C < LE < HE, whereas the increase of As and Zn contents was significant (*p* < 0.05) in the feces of the HE group vs. both C and LE groups. Although the element contents in the feces varied during the experiment, no significant trend was identified for all the elements and their rates. The contents of Cd, Pb, and Zn in the feces of *M. arvalis* increased significantly (*p* < 0.05) in the order of C < LE < HE, documenting enhanced release of these elements from the animal organisms with enhanced uptake of them in the diet. For As, although its contents in the feces of *M. arvalis* tended to a higher content in the LE group compared to C, a statistically (*p* < 0.05) different increase of fecal As was observed only for the HE group, compared to both the LE and C groups. Similar to *A. sylvaticus*, no changes in RE excretion dynamics among groups were shown statistically significant during the experiment.

Result of the present study confirmed transport of soil- and plant-bound risk elements to animal body via dietary intake. These relationships were previously proven in real conditions by Tersago et al. (2004), where the As, Cd, and Pb contents in the *A. sylvaticus* liver decreased with decreasing soil element contents, or in experiment conducted on laboratory rats by Száková et al. (2012), who showed increasing contents of REs in animal tissues related to the contamination level of soils added to the diet. The comprehensive assessment of Cd uptake by *M. arvalis* identified by Veltman et al. (2007) showed a good correlation between Cd concentration in the animal body and in the diet, and the correlation of the animal Cd contents with the Cd content in the soil was proven as well. Similarly, Shore (1995) reported (i) significant correlations between Pb and Cd contents in the tissues of *A. sylvaticus* and *M. agrestis* and the contents of these elements in soil, and (ii) significant relationships between the element contents in the tissues of the individual animal species. According to the latter-mentioned author, estimation of the environmental risk of the soil-bound RE based on the element accumulation in the tissues of one species could be applied to other small terrestrial rodents living in the same area. However, the results of our study provide evidence that the toxicokinetics of individual elements may differ to some extent between individual rodent species.

## Conclusions

The results of this study indicate a generally similar response of both wild species (*M. arvalis* and *A. sylvaticus*) to the elevated RE contents in the diet amended with contaminated soil, confirming these wild rodents as suitable models for RE biomonitoring in ecosystems. However, our data have highlighted some distinction in As toxicokinetics in wood mice when compared to that of field vole and significant variability in RE accumulation related to sex during high exposure levels. Therefore, unlike the common vole, the wood mice may not be a suitable bioindicator of arsenic contamination, and internal factors such as sex of the specimens have to be considered to eliminate bias in risk element monitoring studies conducted on small terrestrial rodents.

## Supplementary information

ESM 1: Supplement – S2: Correlation plots for particular REs in liver (L) and kidney (K). Supplementary table S1a. Individual morphological characteristics of experimental animals. Table S1b. Individual morphological characteristics of experimental animals (DOCX 38 kb)

## Data Availability

The datasets used and analyzed during the current survey are available from the corresponding author on reasonable request.
