# Supplementary material for: The oral bioavailability of soil-borne risk elements for small terrestrial mammals: Microtus arvalis (Pallas, 1778) and Apodemus sylvaticus L. and its implication in environmental studies
Source: Environ Sci Pollut Res Int. 2023 Mar 21;30(22):62397–409. doi: 10.1007/s11356-023-26437-z (PMC10167179; doi:10.1007/s11356-023-26437-z)
Supplement: Supplementary file 1 — Supplement – S2: Correlation plots for particular REs in liver (L) and kidney (K). Supplementary table S1a. Individual morphological characteristics of experimental animals. Table S1b. Individual morphological characteristics of experimental animals (DOCX 38 kb) [file 11356_2023_26437_MOESM1_ESM.docx]

Supplement – S2: Correlation plots for particular REs in liver (L) and kidney (K)

| ***Apodemus sylvaticus*** | | | | |  |  |  |  | ***Microtus arvalis*** | | | |  |  |  |  |  |  |  |  |
| --- | --- | --- | --- | --- | --- | --- | --- | --- | --- | --- | --- | --- | --- | --- | --- | --- | --- | --- | --- | --- |
|  |  |  |  |  |  |  |  |  |  |  |  |  |  |  |  |  |  |  |  |  |
| C | Zn - K | Zn - L | As - K | As - L | Cd - K | Cd - L | Pb -K |  | C | Zn - K | Zn - L | As - K | As - L | Cd - K | Cd - L | Pb - K |  |  |  |  |
| Zn - L | 1 |  |  |  |  |  |  |  | Zn - L | 1 |  |  |  |  |  |  |  | 1 | **1** |  |
| As - K | 0 | 0 |  |  |  |  |  |  | As - K | 1 | 1 |  |  |  |  |  |  | 1 |  |  |
| As - L | 0 | 0 | 0 |  |  |  |  |  | As - L | 0 | 0 | 0 |  |  |  |  |  | 1 |  |  |
| Cd - K | 0 | 0 | 0 | 0 |  |  |  |  | Cd - K | 0 | 0 | 0 | 0 |  |  |  |  | 1 |  |  |
| Cd - L | 0 | 0 | 0 | 0 | 1 |  |  |  | Cd - L | 0 | 0 | 0 | 0 | 1 |  |  |  | 1 |  |  |
| Pb -K | 0 | 0 | 0 | 0 | 0 | 0 |  |  | Pb - K | 1 | 0 | 0 | 0 | 1 | 1 |  |  | 1 | **0.5** |  |
| Pb - L | 0 | 0 | 0 | 0 | 0 | 0 | 1 |  | Pb - L | 0 | -1 | 0 | 1 | 0 | 0 | 0 |  | 0 |  |  |
|  |  |  |  |  |  |  |  |  |  |  |  |  |  |  |  |  |  | 0 |  |  |
| LE | Zn - K | Zn - L | As - K | As - L | Cd - K | Cd - L | Pb -K |  | LE | Zn - K | Zn - L | As - K | As - L | Cd - K | Cd - L | Pb - K |  | 0 |  |  |
| Zn - L | 0 |  |  |  |  |  |  |  | Zn - L | 0 |  |  |  |  |  |  |  | 0 |  |  |
| As - K | 0 | 1 |  |  |  |  |  |  | As - K | 0 | 0 |  |  |  |  |  |  | 0 | **0** |  |
| As - L | 0 | 0 | 0 |  |  |  |  |  | As - L | 0 | 0 | 0 |  |  |  |  |  | 0 |  |  |
| Cd - K | 0 | 1 | 1 | 1 |  |  |  |  | Cd - K | 1 | -1 | 0 | 1 |  |  |  |  | 0 |  |  |
| Cd - L | 0 | 1 | 1 | 1 | 1 |  |  |  | Cd - L | 0 | 0 | 0 | 0 | 0 |  |  |  | 0 |  |  |
| Pb -K | 1 | 0 | -1 | 1 | 0 | 0 |  |  | Pb - K | 0 | -1 | 0 | 0 | 1 | 0 |  |  | 0 |  |  |
| Pb - L | 0 | 1 | 0 | 0 | 1 | 1 | 1 |  | Pb - L | 0 | 0 | 0 | 0 | 0 | 0 | 0 |  | -1 |  |  |
|  |  |  |  |  |  |  |  |  |  |  |  |  |  |  |  |  |  | -1 | **-0.5** |  |
| HE | Zn - K | Zn - L | As - K | As - L | Cd - K | Cd - L | Pb -K |  | HE | Zn - K | Zn - L | As - K | As - L | Cd - K | Cd - L | Pb - K |  | -1 |  |  |
| Zn - L | 0 |  |  |  |  |  |  |  | Zn - L | 1 |  |  |  |  |  |  |  | -1 |  |  |
| As - K | 0 | -1 |  |  |  |  |  |  | As - K | -1 | 0 |  |  |  |  |  |  | -1 |  |  |
| As - L | 0 | 1 | 0 |  |  |  |  |  | As - L | -1 | 0 | 0 |  |  |  |  |  | -1 | **-1** |  |
| Cd - K | 0 | 0 | -1 | 0 |  |  |  |  | Cd - K | 0 | 0 | 0 | 0 |  |  |  |  |  |  |  |
| Cd - L | 0 | 1 | -1 | 0 | 1 |  |  |  | Cd - L | 0 | 1 | 0 | 0 | 1 |  |  |  | L = liver | | |
| Pb -K | 0 | 1 | -1 | 0 | 1 | 1 |  |  | Pb - K | 1 | 1 | 0 | 0 | 1 | 1 |  |  | K = kidney | | |
| Pb - L | 0 | 1 | -1 | 1 | 1 | 1 | 1 |  | Pb - L | 1 | 1 | 0 | 0 | 0 | 1 | 1 |  |  |  |  |

Supplementary table S1a. Individual morphological characteristics of experimental animals

| **Nr** | ***Species*** | **Diet** | **Sex** | **Initial body weight (g)** | **Final body weight (g)** | **Body lenght (mm)** | **Tail lenght (mm)** | **Pawn lenght (mm)** | **Ear lenght (mm)** |
| --- | --- | --- | --- | --- | --- | --- | --- | --- | --- |
| 1 | *Microtus arvalis* | HE | F | 17.1 | 21.0 | 89.6 | 24.6 | 13.0 | 8.9 |
| 2 | *Microtus arvalis* | HE | F | 18.5 | 21.2 | 90.0 | 30.1 | 13.3 | 10.0 |
| 3 | *Microtus arvalis* | HE | F | 19.2 | 22.9 | 91.2 | 30.9 | 13.6 | 10.0 |
| 4 | *Microtus arvalis* | HE | F | 19.6 | 24.8 | 92.2 | 31.0 | 14.0 | 10.6 |
| 5 | *Microtus arvalis* | HE | F | 21.6 | 25.9 | 95.0 | 31.9 | 14.2 | 10.6 |
| 6 | *Microtus arvalis* | HE | M | 23.4 | 27.7 | 96.5 | 32.3 | 14.8 | 10.8 |
| 7 | *Microtus arvalis* | HE | F | 26.0 | 30.4 | 98.2 | 32.5 | 14.9 | 10.9 |
| 8 | *Microtus arvalis* | HE | F | 26.6 | 30.7 | 98.7 | 33.0 | 15.0 | 11.0 |
| 9 | *Microtus arvalis* | HE | M | 27.2 | 31.6 | 99.0 | 33.2 | 15.1 | 11.2 |
| 10 | *Microtus arvalis* | HE | M | 27.4 | 32.1 | 101.6 | 33.7 | 15.2 | 11.2 |
| 11 | *Microtus arvalis* | HE | M | 28.5 | 32.5 | 103.6 | 33.8 | 15.5 | 11.6 |
| 12 | *Microtus arvalis* | HE | M | 28.6 | 35.6 | 104.0 | 35.4 | 16.0 | 11.6 |
| 13 | *Microtus arvalis* | HE | M | 30.3 | 37.2 | 108.0 | 35.7 | 16.0 | 12.0 |
| 14 | *Microtus arvalis* | HE | M | 44.4 | 45.9 | 110.8 | 36.0 | 17.0 | 12.9 |
| 15 | *Microtus arvalis* | HE | M | 44.6 | 51.8 | 124.0 | 43.0 | 19.0 | 13.0 |
| 16 | *Microtus arvalis* | LE | F | 16.4 | 20.6 | 83.1 | 24.5 | 13.0 | 9.0 |
| 17 | *Microtus arvalis* | LE | M | 20.2 | 22.9 | 93.9 | 26.7 | 13.0 | 9.1 |
| 18 | *Microtus arvalis* | LE | F | 21.3 | 23.3 | 95.0 | 28.3 | 13.3 | 9.8 |
| 19 | *Microtus arvalis* | LE | F | 21.4 | 23.7 | 100.5 | 29.2 | 13.5 | 10.5 |
| 20 | *Microtus arvalis* | LE | F | 24.4 | 25.3 | 100.9 | 31.0 | 13.9 | 10.6 |
| 21 | *Microtus arvalis* | LE | M | 25.2 | 26.1 | 101.0 | 32.0 | 14.0 | 10.6 |
| 22 | *Microtus arvalis* | LE | F | 26.0 | 32.4 | 101.0 | 32.8 | 14.4 | 10.9 |
| 23 | *Microtus arvalis* | LE | F | 27.0 | 34.5 | 102.0 | 33.0 | 14.5 | 11.0 |
| 24 | *Microtus arvalis* | LE | F | 27.9 | 34.5 | 102.0 | 34.0 | 14.9 | 11.0 |
| 25 | *Microtus arvalis* | LE | M | 32.1 | 35.5 | 107.5 | 34.0 | 15.0 | 11.0 |
| 26 | *Microtus arvalis* | LE | M | 32.9 | 38.5 | 108.0 | 36.0 | 15.0 | 11.0 |
| 27 | *Microtus arvalis* | LE | M | 33.2 | 41.8 | 108.0 | 37.0 | 15.0 | 11.1 |
| 28 | *Microtus arvalis* | LE | M | 36.3 | 42.3 | 109.0 | 37.2 | 16.6 | 11.5 |
| 29 | *Microtus arvalis* | LE | M | 37.2 | 42.8 | 115.0 | 39.0 | 17.0 | 12.2 |
| 30 | *Microtus arvalis* | LE | M | 46.0 | 54.9 | 118.0 | 43.0 | 18.0 | 13.0 |
| 31 | *Microtus arvalis* | CO | F | 17.3 | 20.7 | 92.2 | 24.6 | 13.3 | 10.0 |
| 32 | *Microtus arvalis* | CO | F | 18.7 | 20.8 | 90.0 | 32.5 | 14.3 | 10.7 |
| 33 | *Microtus arvalis* | CO | F | 22.8 | 25.3 | 89.0 | 30.5 | 15.3 | 10.8 |
| 34 | *Microtus arvalis* | CO | M | 30.6 | 28.1 | 97.6 | 26.5 | 14.5 | 11.0 |
| 35 | *Microtus arvalis* | CO | M | 30.7 | 40.9 | 99.8 | 29.9 | 13.6 | 11.1 |
| 36 | *Microtus arvalis* | CO | M | 34.8 | 41.6 | 109.0 | 35.0 | 16.0 | 12.0 |
| 37 | *Microtus arvalis* | CO | F | 39.9 | 44.0 | 109.0 | 34.0 | 15.0 | 12.0 |
| 38 | *Microtus arvalis* | CO | M | 44.4 | 49.2 | 120.0 | 35.0 | 16.0 | 12.1 |
|  |  |  |  |  |  |  |  |  |  |

Table S1b. Individual morphological characteristics of experimental animals

| **Nr** | ***Species*** | **Diet** | **Sex** | **Initial body weight (g)** | **Final body weight (g)** | **Body lenght (mm)** | **Tail lenght (mm)** | **Pawn lenght (mm)** | **Ear lenght (mm)** |
| --- | --- | --- | --- | --- | --- | --- | --- | --- | --- |
| 1 | *Apodemus sylvaticus* | HE | F | 16.5 | 19.9 | 86.1 | 74.3 | 21.7 | 13.3 |
| 2 | *Apodemus sylvaticus* | HE | F | 17.1 | 21.4 | 90.0 | 88.0 | 19.6 | 14.3 |
| 3 | *Apodemus sylvaticus* | HE | F | 17.1 | 21.5 | 92.0 | 82.0 | 21.0 | 13.5 |
| 4 | *Apodemus sylvaticus* | HE | F | 19.8 | 22.1 | 87.6 | 81.4 | 20.3 | 14.4 |
| 5 | *Apodemus sylvaticus* | HE | M | 24.2 | 25.1 | 95.4 | 88.0 | 21.0 | 13.9 |
| 6 | *Apodemus sylvaticus* | HE | M | 24.7 | 27.4 | 94.0 | 98.0 | 22.0 | 17.0 |
| 7 | *Apodemus sylvaticus* | HE | M | 27.0 | 30.1 | 98.0 | 83.0 | 20.0 | 15.0 |
| 8 | *Apodemus sylvaticus* | LE | F | 16.3 | 18.9 | 92.3 | 82.0 | 21.0 | 11.4 |
| 9 | *Apodemus sylvaticus* | LE | F | 18.8 | 20.9 | 91.8 | 70.9 | 20.0 | 14.4 |
| 10 | *Apodemus sylvaticus* | LE | F | 19.0 | 24.3 | 90.0 | 84.0 | 20.5 | 15.5 |
| 11 | *Apodemus sylvaticus* | LE | M | 20.5 | 24.6 | 97.4 | 84.7 | 20.9 | 15.7 |
| 12 | *Apodemus sylvaticus* | LE | F | 23.5 | 26.9 | 95.0 | 76.0 | 21.0 | 16.0 |
| 13 | *Apodemus sylvaticus* | LE | M | 24.9 | 27.7 | 98.0 | 82.0 | 18.0 | 17.0 |
| 14 | *Apodemus sylvaticus* | LE | M | 26.3 | 32.9 | 93.7 | 90.6 | 20.1 | 17.6 |
| 15 | *Apodemus sylvaticus* | CO | F | 18.3 | 20.0 | 90.3 | 86.4 | 21.3 | 16.8 |
| 16 | *Apodemus sylvaticus* | CO | F | 19.7 | 20.8 | 86.2 | 84.5 | 20.8 | 16.4 |
| 17 | *Apodemus sylvaticus* | CO | F | 20.0 | 21.2 | 95.0 | 87.0 | 18.0 | 15.4 |
| 18 | *Apodemus sylvaticus* | CO | F | 22.6 | 21.4 | 95.9 | 79.0 | 20.0 | 13.3 |
| 19 | *Apodemus sylvaticus* | CO | M | 23.3 | 26.6 | 93.7 | 89.5 | 21.8 | 14.2 |
| 20 | *Apodemus sylvaticus* | CO | M | 24.7 | 28.7 | 99.8 | 97.9 | 20.8 | 16.2 |
| 21 | *Apodemus sylvaticus* | CO | M | 26.7 | 30.7 | 93.8 | 98.0 | 22.5 | 17.6 |
